# Does the Panoramic Radiography Have the Power to Identify the Gonial Angle in Orthodontics?

**DOI:** 10.1100/2012/219708

**Published:** 2012-12-13

**Authors:** Rıdvan Okşayan, Ali Murat Aktan, Oral Sökücü, Esin Haştar, Mehmet Ertuğrul Ciftci

**Affiliations:** ^1^Department of Orthodontics, Faculty of Dentistry, Gaziantep University, 27310 Gaziantep, Turkey; ^2^Department of Dentomaxillofacial Radiology, Faculty of Dentistry, Gaziantep University, 27310 Gaziantep, Turkey

## Abstract

*Purpose*. The objective of this study was to assess gonial angle under the angle classification by comparing panoramic radiograph and lateral cephalometric radiograph. *Materials and Methods*. 49 patients (25 males, 24 females) with an age range of 12–29 years participated in the present study. Subjects were retrospectively selected among those categorised as skeletal and dental Class I, II, and III malocclusion group. Using lateral cephalometric radiograph, mandibular and ramal planes were drawn and based on these planes. Gonial angle was determined from two tangents which were drawn from the inferior border of the mandible and posterior borders of the condyle and ramus of both sides in the panoramic radiographs. Multiple comparison tests (ANOVA) were used to determine differences between the three angle groups. *Results*. There were no significant differences between Class I, II, and III malocclusion group values of gonial angles determined by lateral cephalometric radiograph and panoramic radiographs (*P* > 0.05). *Conclusion*. Panoramic radiograph results were shown to be as reliable as lateral cephalometric radiograph in all angle classifications. Panoramic radiography can be used as an alternative radiographic technique to detect gonial angle in orthodontic patients.

## 1. Introduction

The panoramic radiographs were used in orthodontic practice to provide information about axial inclinations, maturation periods, and surrounding tissues of the teeth. Lateral cephalogram is another radiographic technique used when cephalometric measurements are made. The gonial angle is widely used in orthodontic cephalogram tracing. This angle is the angle between an imaginary tangential line along the inferior border of the mandible and along the posterior border of the mandibular ramus [1]. 

The morphology of the superficial masseter muscle in the gonion region differs between dentulous and edentulous subjects [2]. The authors showed that mandibular bone after tooth extraction includes a chronic and progressive resorption of the residual ridge and may also present as a widening of the gonial angle. 

In orthodontics, gonial angle is used commonly to determine the rotation of the mandible. The gonial angle is a significant indicator to diagnose the growth pattern of patients. The downward and backward rotation is called as a high angle in patients who showed increase of gonial angle. Contrary to this, upward and forward direction of mandible called as a low angle that appears decreased of gonial angle on patients [3]. This is one of the PARAMETER affects the extraction of teeth on Class II patients [4]. In Class III patients this gonial angle may affect the treatment approach, and helps to decide underwent surgery or not [5].

Although, Mattila et al. [6] stated that gonial angle can be determined from panoramic radiography with the same degree of accuracy as from lateral cephalogram, Fischer-Brandies et al. [7] preferred only the lateral cephalogram in determining gonial angle. Furthermore, some investigators showed a great individual variations in gonial angle distortion which was affected by age and in different types of malocclusion [8, 9]. To our knowledge, there was no study regarding the variations in gonial angle and the whole malocclusions in orthodontics. Therefore, the aim of this study was to determine the gonial angle under the Angle classification by comparing the panoramic radiographs and lateral cephalograms.

## 2. Materials and Methods

A total of 63 patients (28 males, 35 females; age range, 12–29 years) who attended our clinic for orthodontic treatment participated in the present study. The subject participation in this study was retrospectively selected among patients that indicate both skeletally and dentally Cl I, II, and III relationship. The control group was selected from subjects who did not receive orthodontic treatment previously, has Class I skeletal feature, and has an ideal occlusion. The cephalometric data included SNA, SNB, ANB, SN-GOGN, and SF-GON measurements of the selected patients and they confirmed these classifications (Table 1). The radiographic data included lateral cephalometric and panoramic radiographs. The radiographs were taken with the same digital machine (Sirona, XG 3, München, Germany). The criteria for selection of patients radiographs had to be high quality and sharpness, and all radiographs had to be taken by the same apparatus and same technician, and patients in natural head position. The subjects were skeletally classified by evaluating cephalometric norms, particularly ANB angle, on the lateral cephalograms in the sagittal plane. No subgroups were constructed among Class II malocclusion cases.

**Figure 1:**
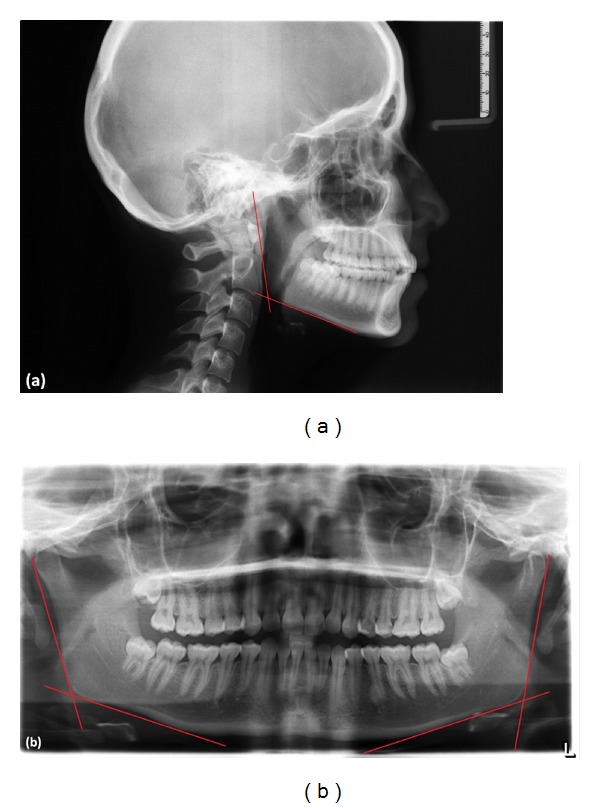
Measurement of the gonial angle in LCR (a) and PR (b).

In lateral cephalograms, mandibular and ramal planes were drawn and based on these planes, and gonial angle was determined. In panoramic radiographs, the gonial angle was determined from two tangents which were drawn from the inferior border of the mandible and posterior borders of condyle and ramus of both sides (Figures 1(a) and 1(b)).

### 2.1. Statistical Analysis

Descriptive statistics for each measurement were calculated. Exploratory analysis (Klomogorov-Smirnov test) revealed that data was normally distributed. Since the data were normally distributed, multiple comparison tests (ANOVA) and Tukey tests were used to determine differences among and between the four groups. Within the group, changes were assessed using a paired *t*-test. All data were analyzed using SPSS version 17.

## 3. Results

The study group consisted of 63 subjects (28 males, 35 females, mean age; 17, 37 ± 3.98) with various malocclusions and was divided into four subgroups according to the Angle-based malocclusion type as follows: Cl I 21 subjects (14 males, 7 females, mean age; 16, 48 ± 2, 87), Cl II 14 subjects (6 males, 8 females, mean age; 14, 00 ± 0.88), Cl III 14 subjects (5 males, 9 females; mean age; 18, 00 ± 4, 67), and the control group 14 subjects (3 males, 11 females; mean age; 21, 43 ± 2, 97). 

The mean values of the gonial angle in lateral cephalogram and panoramic radiographs according to malocclusion types were shown in Table 2. In Table 3, Tukey test shows the differences in subgroups. There were no significant differences among the Class I, II, and III malocclusions groups' values of gonial angles determined by lateral cephalograms and panoramic radiographs. However, the result of the study showed that the gonial angle of control group was significantly different from Class I, II, and III malocclusions.

There were no significant differences between males and females subjects on the determination of the gonial angle by lateral cephalograms and panoramic radiographs. The gonial angle in females was 124.40° and that in males 123.34° with no statistically significant differences between the two genders.

In panoramic radiographs, the mean value of the right gonial angle was 123° (±6°) and the mean value of the left gonial angle was 123° (±5°). There was no significant difference between the right side and left side in panoramic measurement for all groups.

The differences between gonial angle measurements in lateral cephalogram and panoramic radiography were found 0.04 in the right side and 0.02 in the left side. Variation in the two radiographic techniques showed no statistically significant differences (*P* > 0.169 in the right side and *P* > 0.889 in the left side). 

## 4. Discussion

In orthodontics, many orthodontists believe that only lateral cephalogram is useful to define the gonial angle [7]. There are several studies that suggested panoramic radiography to determine the gonial angle [1, 10, 11]. However many of them purposed to evaluate changes PAGE 2 evaluate the gonial angle changes in dentulous or/and edentulous states [11, 12]. In orthodontics, the reliability of panoramic radiography was evaluated by Shahabi only on Angle Class I patients [9]. We aimed to observe the gonial angle on all Angle classifications. This is the first study in the literature that appears to compare the measurement of gonial angle on the different orthodontic malocclusions with panoramic radiographs and lateral cephalograms.

According to the result of this study, control and study groups showed similar results on both radiographic techniques. That result revealed that the gonial angle on control group was significantly less than other all orthodontic malocclusion groups. All the malocclusion groups showed differences with control group on both radiographic techniques. This finding suggested us that a panoramic radiograph is successful in determining gonial angle as well as lateral cephalogram. In Class I patients Shahabi et al. [9] supported our findings and declared that it may be a better choice to use panoramic radiographs than a lateral cephalogram for determination of the gonial angle. Our results did not show significant differences between two radiographic methods. 

Class III patients is important in orthodontics because we often need to give decision on patients to go on surgery or not. Tahmina et al. [5] found that the increase of gonial angle is one of the marker need surgery on Class III patient conservative. The increase of this landmark showed unstable results after orthodontic treatment. On the other hand, the patients especially that followed up by orthodontic clinics may need per year lateral cephalogram for evaluating this point. Getting only panoramic radiographs from these patients may eliminate extra apply radiation doses and costs. Therefore it is important in orthodontics to use the panoramic films as a record. Our results showed that the panoramic films are reliable as lateral cephalograms in orthodontics.

In the present study, the left and right gonial angles obtained by panoramic radiography were not statistically different. These findings coincided with the results of previous studies [9, 13]. The gonial angle was obtained by panoramic radiography −0.19°, −1.04°, 0.48°, and 0.68° on the right sides in the C I, CII, CIII, and control group less than that of lateral cephalograms, respectively. In addition the differences on the gonial angle measurements between left sides lateral and cephalograms were found as −0.29°, 0.28°, 0.23°, and −0.17°. These findings showed that there were no statistically significant differences between the two radiographs on both sides, which consisted of results reported by Shahabi et al. [9]. However, Fisher-Brandies found gonial angle on panoramic radiograph 2.2–3.6 degrees less than lateral cephalograms, also these results showed significant difference between two radiographs [7]. Shahabi et al. [9] declared that the disparity in the results could be because of the type of malocclusion and age of the samples while their study was performed in adults with Class I malocclusion. Although, their findings consisted of the present findings, the current study was differentiated from their study since the present study was constructed by either adult or adolescent patients with all kinds of malocclusion. Another result of the present study was that there was no statistically significant gender difference in the gonial angle determined by the two radiographic techniques, which was in agreement with the results of the previous studies [14]. Ohm and Silness, stated that the gender had little effect on the size of the gonial angle [15]. Therefore it is suggested that the effect of the age and gender on the gonial angle was so limited or absent [16].

Analysis of the two techniques suggests that gonial angle parameters deliver reliable results using PR. This allows a reduction in patient radiation doses before or during orthodontic treatment.

## Figures and Tables

**Table 1:** Maximum, minimum, least square means, and standard deviation of the means for patients.

Measurements		*N*	Mean age	Sd	Mean	Sd	Min	Max
SNA	Class I	21	16.48	2.87	78.10	2.022	74	82
	Class II	14	14.00	0.88	79.71	3.583	75	87
	Class III	14	18.00	4.67	77.86	3.394	72	84
	**Total**	**49**	**16.20**	**3.45**	**78.49**	**2.987**	**72**	**87**

SNB	Class I	21	16.48	2.87	75.81	2.118	72	80
	Class II	14	14.00	0.88	74.07	3.435	70	82
	Class III	14	18.00	4.67	80.39	4.063	73	88
	**Total**	**49**	**16.20**	**3.45**	**76.62**	**3.984**	**70**	**88**

ANB	Class I	21	16.48	2.87	2.14	1.014	1	4
	Class II	14	14.00	0.88	5.39	1.212	5	9
	Class III	14	18.00	4.67	−2.69	2.391	−9	0
	**Total**	**49**	**16.20**	**3.45**	**1.69**	**3.468**	**−9**	**9**

SN-GOGN	Class I	21	16.48	2.87	33.48	4.831	25	43
	Class II	14	14.00	0.88	34.18	7.505	23	43
	Class III	14	18.00	4.67	34.11	7.761	23	52
	**Total**	**49**	**16.20**	**3.45**	**33.86**	**6.435**	**23**	**52**

SF-GON	Class I	21	16.48	2.87	124.57	5.427	115	137
	Class II	14	14.00	0.88	123.64	6.547	112	135
	Class III	14	18.00	4.67	126.86	7.004	116	139
	**Total**	**49**	**16.20**	**3.45**	**124.96**	**6.228**	**112**	**139**

**Table 2:** ANOVA test revealed *P* values related to the gonial angle in LCR and PR according to the right and left sides.

	Group	*N*	Mean	Sd	Among groups *P*
Panoramic (Right)	Class I	21	125.95	6.72	
	Class II	14	124.18	6.54	0.422
	Class III	14	127.97	9.47	

Lateral (Right)	Class I	21	125.75	7.11	
	Class II	14	123.02	8.40	0.219
	Class III	14	128.56	9.63	

Panoramic (Left)	Class I	21	125.40	6.59	
	Class II	14	123.91	6.14	0.834
	Class III	14	125.18	9.51	

Lateral (Left)	Class I	21	124.91	6.98	
	Class II	14	124.35	7.07	0.928
	Class III	14	125.51	9.88	

**Table 3:** The gonial degree changes in PR for both sides.

		*N*	Mean	Sd	*P*
	Class I	21	−0.18	2.59	
Right	Class II	14	−1.04	2.94	0.24
	Class III	14	0.47	0.78	

	Class I	21	−0.29	3.46	
Left	Class II	14	0.28	2.81	0.79
	Class III	14	0.23	1.17	
